# Religion, Spirituality, and Health: The Research and Clinical Implications

**DOI:** 10.5402/2012/278730

**Published:** 2012-12-16

**Authors:** Harold G. Koenig

**Affiliations:** ^1^Departments of Medicine and Psychiatry, Duke University Medical Center, P.O. Box 3400, Durham, NC 27705, USA; ^2^Department of Medicine, King Abdulaziz University, Jeddah 21413, Saudi Arabia

## Abstract

This paper provides a concise but comprehensive review of research on religion/spirituality (R/S) and both mental health and physical health. It is based on a systematic review of original data-based quantitative research published in peer-reviewed journals between 1872 and 2010, including a few seminal articles published since 2010. First, I provide a brief historical background to set the stage. Then I review research on R/S and mental health, examining relationships with both positive and negative mental health outcomes, where positive outcomes include well-being, happiness, hope, optimism, and gratefulness, and negative outcomes involve depression, suicide, anxiety, psychosis, substance abuse, delinquency/crime, marital instability, and personality traits (positive and negative). I then explain how and why R/S might influence mental health. Next, I review research on R/S and health behaviors such as physical activity, cigarette smoking, diet, and sexual practices, followed by a review of relationships between R/S and heart disease, hypertension, cerebrovascular disease, Alzheimer's disease and dementia, immune functions, endocrine functions, cancer, overall mortality, physical disability, pain, and somatic symptoms. I then present a theoretical model explaining how R/S might influence physical health. Finally, I discuss what health professionals should do in light of these research findings and make recommendations in this regard.

## 1. Historical Background and Introduction

Religion, medicine, and healthcare have been related in one way or another in all population groups since the beginning of recorded history [1]. Only in recent times have these systems of healing been separated, and this separation has occurred largely in highly developed nations; in many developing countries, there is little or no such separation. The history of religion, medicine, and healthcare in developed countries of the West, though, is a fascinating one. The first hospitals in the West for the care of the sick in the general population were built by religious organizations and staffed by religious orders. Throughout the Middle Ages and up through the French Revolution, physicians were often clergy. For hundreds of years, in fact, religious institutions were responsible for licensing physicians to practice medicine. In the American colonies, in particular, many of the clergy were also physicians—often as a second job that helped to supplement their meager income from church work.

Care for those with *mental health* problems in the West also had its roots within monasteries and religious communities [2]. In 1247, the Priory of St. Mary of Bethlehem was built in London on the Thames River [3]. Originally designed to house “distracted people,” this was Europe's (and perhaps the world's) first mental hospital. In 1547, however, St. Mary's was torn down and replaced by Bethlehem or Bethlem Hospital [4]. Over the years, as secular authorities took control over the institution, the hospital became famous for its inhumane treatment of the mentally ill, who were often chained [5], dunked in water, or beaten as necessary to control them. In later years, an admission fee (2 pence) was charged to the general public to observe the patients abusing themselves or other patients [4]. The hospital eventually became known as “bedlam” (from which comes the word used today to indicate a state of confusion and disarray).

In response to the abuses in mental hospitals, and precipitated by the death of a Quaker patient in New York asylum in England, an English merchant and devout Quaker named William Tuke began to promote a new form of treatment of the mentally ill called “moral treatment.” In 1796, he and the Quaker community in England established their own asylum known as the York Retreat [6]. Not long after this, the Quakers brought moral treatment to America, where it became the dominant form of psychiatric care in that country [6]. Established in Philadelphia by the Quakers in 1813, “Friends Hospital” (or Friends Asylum) became the first private institution in the United States dedicated solely to the care of those with mental illness [7]. Psychiatric hospitals that followed in the footsteps of Friends Asylum were the McLean Hospital (established in 1818 in Boston, and now associated with Harvard), the Bloomingdale Asylum (established in 1821 in New York), and the Hartford Retreat (established in 1824 in Connecticut)—all modeled after the York Retreat and implementing moral treatment as the dominant therapy.

It was not until modern times that religion and psychiatry began to part paths. This separation was encouraged by the psychiatrist Sigmund Freud. After being “introduced” to the neurotic and hysterical aspects of religion by the famous French neurologist Jean Charcot in the mid-1880s, Freud began to emphasize this in a widely read series of publications from 1907 through his death in 1939. Included among these were *Religious Acts and Obsessive Practices* [8], *Psychoanalysis and Religion* [9], *Future of an Illusion* [10], and *Moses and Monotheism* [11]. These writings left a legacy that would influence the practice of psychiatry—especially psychotherapy—for the rest of the century and lead to a true schism between religion and mental health care. That schism was illustrated in 1993 by a systematic review of the religious content of DSM-III-R, which found nearly one-quarter of all cases of mental illness being described using religious illustrations [12]. The conflict has continued to the present day. Consider recent e-letters in response to two articles published in *The Psychiatrist* about this topic [13, 14] and an even more recent debate about the role of prayer in psychiatric practice [15]. This conflict has manifested in the clinical work of many mental health professionals, who have generally ignored the religious resources of patients or viewed them as pathological. Consider that a recent national survey of US psychiatrists found that 56% said they never, rarely, or only sometimes inquire about religious/spiritual issues in patients with depression or anxiety [16]. Even more concerning, however, is that the conflict has caused psychiatrists to avoid conducting research on religion and mental health. This explains why so little is known about the relationship between religious involvement and severe mental disorders (see *Handbook of Religion and Health*) [17].

Despite the negative views and opinions held by many mental health professionals, research examining religion, spirituality, and health has been rapidly expanding—and most of it is occurring outside the field of psychiatry. This research is being published in journals from a wide range of disciplines, including those in medicine, nursing, physical and occupational therapy, social work, public health, sociology, psychology, religion, spirituality, pastoral care, chaplain, population studies, and even in economics and law journals. Most of these disciplines do not readily communicate with each another, and their journal audiences seldom overlap. The result is a massive research literature that is scattered throughout the medical, social, and behavioral sciences.

**Figure 1:**
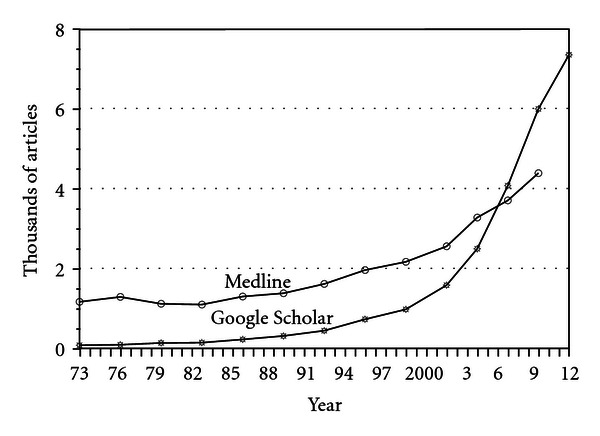
Religion spirituality and health articles published per 3-year period (noncumulative) Search terms: religion, religious, religiosity, religiousness, and spirituality (conducted on 8/11/12; projected to end of 2012).

To get a sense of how rapidly the research base is growing see Figure 1. The graphs plot the number of studies published in peer-reviewed journals during every noncumulative 3-year period from 1971 to 2012. Note that about 50% of these articles are reports of original research with quantitative data, whereas the other 50% are qualitative reports, opinion pieces, reviews, or commentaries. Google Scholar presents a more comprehensive picture since it includes studies published in both Medline and non-Medline journals. These graphs suggest that the volume of research on R/S and health has literally exploded since the mid-1990s.

## 2. Definitions

Before summarizing the research findings, it is first necessary to provide definitions of the words religion and spirituality that I am using. There is much controversy and disagreement concerning definitions in this field, particularly over the term “spirituality,” and space here does not allow a full discussion of these complex issues. For an in depth discussion, including an exploration of contamination and confounding in the measurement of spirituality, I refer the reader to other sources [18–20]. Here are the definitions we provided in the *Handbook*.

“[Religion] Involves beliefs, practices, and rituals related to the *transcendent*, where the transcendent is God, Allah, HaShem, or a Higher Power in Western religious traditions, or to Brahman, manifestations of Brahman, Buddha, Dao, or ultimate truth/reality in Eastern traditions. This often involves the mystical or supernatural. Religions usually have specific beliefs about life after death and rules about conduct within a social group. Religion is a multidimensional construct that includes beliefs, behaviors, rituals, and ceremonies that may be held or practiced in private or public settings, but are in some way derived from established traditions that developed over time within a community. Religion is also an organized system of beliefs, practices, and symbols designed (a) to facilitate closeness to the transcendent, and (b) to foster an understanding of one's relationship and responsibility to others in living together in a community.” [21].

“Spirituality is distinguished from all other things—humanism, values, morals, and mental health—by its connection to that which is sacred, the *transcendent*. The transcendent is that which is outside of the self, and yet also within the self—and in Western traditions is called God, Allah, HaShem, or a Higher Power, and in Eastern traditions may be called Brahman, manifestations of Brahman, Buddha, Dao, or ultimate truth/reality. Spirituality is intimately connected to the supernatural, the mystical, and to organized religion, although also extends beyond organized religion (and begins before it). Spirituality includes both a search for the transcendent and the discovery of the transcendent and so involves traveling along the path that leads from nonconsideration to questioning to either staunch nonbelief or belief, and if belief, then ultimately to devotion and finally, surrender. Thus, our definition of spirituality is very similar to religion and there is clearly overlap.” [22].

For the research review presented here, given the similarity in my definition of these terms and the fact that spirituality in the research has either been measured using questions assessing religion or by items assessing mental health (thereby contaminating the construct and causing tautological results), I will be using religion and spirituality interchangeably (i.e., R/S).

## 3. Method of the Review

I summarize the research findings between R/S and health first in the area of mental health outcomes, then for health behaviors, and finally for physical health outcomes. The information presented here is based on a systematic review of peer-reviewed original data-based reports published though mid-2010 and summarized in two editions of the *Handbook of Religion and Health* [23, 24]. How these systematic reviews were conducted, however, needs brief explanation. This is particularly true for ratings of study methodology that are used to summarize the findings below.

The systematic review to identify the studies presented in the Handbooks and summarized in this paper was conducted as follows. We utilized a combination of strategies to identify the studies (excluding most reviews or qualitative research). First, we systematically searched online databases (PsycINFO, MEDLINE, etc.) using the search words “religion,” “religiosity,” “religiousness,” and “spirituality” to identify studies on the R/S-health relationship. Second, we asked prominent researchers in the field to alert us to published research they knew about and to send us research that they themselves had conducted. Third, if there were studies cited in the reference lists of the studies located, we tracked down those as well. Using this method, we identified over 1,200 quantitative original data-based publications during the period 1872 to 2000 and 2,100 studies examining the R/S-health relationship from 2000 to 2010. All of these studies are described in the appendices of the two editions of the Handbook. Based on other reviews of the research conducted around this same time period (but more limited), we estimate that our review captured about 75% of the published research. Bear in mind that many, many more qualitative studies have been published on the topic that were not included in this review.

In order to assess the methodological quality of the studies, quality ratings were assigned as follows. Ratings of each of the more than 3,300 studies were made on a scale from 0 (low) to 10 (high) and were performed by a single examiner (HGK) to ensure rating consistency. Scores were determined according to the following eight criteria: study design (clinical trial, prospective cohort, cross-sectional, etc.), sampling method (random, systematic, or convenience), number of R/S measures, quality of measures, quality of mental health outcome measure, contamination between R/S measures and mental health outcomes, inclusion of control variables, and statistical method, based on a scheme adapted from Cooper [25]. Cooper emphasized the definition of variables, validity and reliability of measures, representativeness of the sample (sample size, sampling method, and response rates), research methods (quality of experimental manipulation and adequacy of control group for clinical trials), how well the execution of the study conformed to the design, appropriateness of statistical tests (power, control variables), and the interpretation of results.

To assess the reliability of the ratings, we compared HGK's ratings on 75 studies with the ratings made by an independent outside reviewer (Andrew Futterman, Ph.D., professor of psychology, College of the Holy Cross, a scientist familiar with the scoring criteria and active in the field of R/S-health research). When we examined correlations between HGK and Futterman's ratings, we found them moderately correlated (Pearson *r* = 0.57). Since scores of 7 or higher indicated higher quality studies, we also compared the scores between the two raters in terms of lower (0–6) versus higher (7–10) quality. This was done by dichotomizing scores into two categories (0–6 versus 7–10) and comparing the categories between the two examiners. The kappa of agreement (*κ*) between the two raters was 0.49 (where kappas of 0.40 to 0.75 indicate good agreement [26]). Overall, the raters agreed on whether quality was low or high in 56 of the 75 studies or 75%. I now summarize the results of the systematic review described above.

## 4. Religion, Spirituality, and Mental Health

Approximately 80% of research on R/S and health involves studies on mental health. One would expect stronger relationships between R/S and mental health since R/S involvement consists of psychological, social, and behavioral aspects that are more “proximally” related to mental health than to physical health. In fact, we would not expect any direct or immediate effects of R/S on physical health, other than indirectly through intermediary psychosocial and behavioral pathways. With regard to mental health, we would expect R/S to boost positive emotions and help neutralize negative emotions, hypothesizing that it serves as both a life-enhancing factor and as a coping resource. With regard to the latter, there is both qualitative and quantitative research suggesting that R/S helps people to deal better with adversity, either external adversity (difficult environmental circumstances) or internal adversity (genetic predisposition or vulnerability to mental disorders).

In the present paper, I have chosen to cite original reports as examples of the most rigorous studies in each area based on ratings in the Handbooks (i.e., 7 or higher on 0–10 scale). Cited here are both positive and negative studies reporting significant relationships. For some topics, such as well-being and depression, there are too many high-quality studies to cite, so only a few examples of the best studies are provided. 

### 4.1. Coping with Adversity

In the first edition of the Handbook [27], we identified 110 studies published prior to the year 2000 and 344 studies published between 2000 and 2010 for a total of 454 studies. Among these reports are descriptions of how R/S helped people to cope with a wide range of illnesses or in a variety of stressful situations. These include people dealing with general medical illness [28, 29], chronic pain [30], kidney disease [31], diabetes [32, 33], pulmonary disease [34], cancer [35, 36], blood disorders [37], heart/cardiovascular diseases [38, 39], dental [40] or vision [41] problems, neurological disorders [42], HIV/AIDS [43], systemic lupus erythematosus [44], irritable bowel syndrome [45], musculoskeletal disease [46], caregiver burden [47–49],  psychiatric illness [50, 51], bereavement [52, 53], end-of-life issues [54, 55], overall stress [56–58], natural disasters [59, 60], war [61, 62] or acts of terrorism [63], and miscellaneous adverse life situations [64–66]. In the overwhelming majority of studies, people reported that R/S was helpful.

### 4.2. Positive Emotions

Positive emotions include well-being, happiness, hope, optimism, meaning and purpose, high self-esteem, and a sense of control over life. Related to positive emotions are positive psychological traits such as altruism, being kind or compassionate, forgiving, and grateful.

#### 4.2.1. Well-Being/Happiness

By mid-2010, at least 326 quantitative, peer-reviewed studies had examined relationships with R/S. Of those, 256 (79%) found only significant positive associations between R/S and well-being (including eight studies at a statistical trend level, that is, 0.05 < *P* < 0.10). Only three studies (<1%) reported a significant inverse relationship between R/S and well-being. Of the 120 studies with the highest methodological rigor (7 or higher in quality on the 0–10 scale), 98 (82%) reported positive relationships (including two at a trend level) [67–77] and one study reported a negative relationship (but only at a trend level) [78].

#### 4.2.2. Hope

At least 40 studies have examined relationships with R/S, and of those, 29 (73%) reported only significant positive relationships with degree of hope; no studies found an inverse relationship. Of the six highest quality studies, half found a positive relationship [79–81].

#### 4.2.3. Optimism

We located 32 studies examining relationships with R/S, and of those, 26 (81%) reported significant positive relationships. Of the 11 best studies, eight (73%) reported significant positive relationships [82–85]. Again, as with hope, no studies reported inverse relationships.

#### 4.2.4. Meaning and Purpose

At least 45 studies have examined relationships with R/S, and 42 (93%) reported significant positive relationships. These studies were often in populations where there was a challenge to having meaning and purpose, such as in people with chronic disabling illness. Of the 10 studies with quality ratings of 7 or higher, all 10 reported significant positive associations [86–89].

#### 4.2.5. Self-Esteem

Critics have claimed that R/S adversely affects self-esteem because it emphasizes humility rather than pride in the self [90]. Furthermore, R/S could exacerbate guilt in some for not living up to the high standards of conduct prescribed by religious traditions, resulting in low self-esteem. We found 69 studies that examined associations with R/S, and of those, 42 (61%) found *greater* self-esteem among those who were more R/S and two (3%) reported lower self-esteem. Of the 25 studies with the highest methodological rigor, 17 (68%) reported greater self-esteem [91–98] and two (8%) found worse self-esteem [99, 100]. Not surprisingly, these findings are parallel to those of depression below (in the opposite direction, of course).

#### 4.2.6. Sense of Control

Although one might expect R/S to correlate positively with an external locus of control (i.e., the Transcendent controlling events), and some studies confirm this, the majority of research finds a positive correlation with an internal not an external sense of control. Of 21 studies that have examined these relationships, 13 (61%) found that R/S was related to a greater sense of personal control in challenging life circumstances. Of the nine best studies, four reported significant positive relationships (44%) [101–104] and three report significant negative relationships (33%) [105–107], whereas the two remaining studies reported complex or mixed results (significant positive and negative associations, depending on R/S characteristic). R/S beliefs may provide an indirect sense of control over stressful situations; by believing that God is in control and that prayer to God can change things, the person feels a greater sense of internal control (rather than having to depend on external agents of control, such as powerful other people).

#### 4.2.7. Positive Character Traits

With regard to character traits, the findings are similar to those with positive emotions. With regard to altruism or frequency of volunteering, 47 studies have examined relationships with R/S. Of those, 33 (70%) reported significant associations, whereas five (11%) found less altruism among the more R/S; of the 20 best studies, 15 (75%) reported positive relationships [108–113] and two (10%) found negative associations [114, 115] (both concerning organ donations, which some religions prohibit). With regard to forgiveness, 40 studies have examined correlations with R/S, and 34 (85%) reported significant positive relationships and no studies found negative associations. Among the 10 highest quality studies, seven (70%) reported greater forgiveness among the more R/S [116–119], a finding that recent research has supported [120]. Regarding gratefulness, five of five studies found positive associations with R/S [121, 122], and with regard to kindness/compassion, three of three studies reported significant positive relationship with R/S [123]. Admittedly, all of the studies measuring character traits above depend on self-report.

### 4.3. Depression

As with self-esteem, mental health professionals have argued that R/S might increase guilt by focusing on sin and could thus lead to depression. Again, however, this has not been found in the majority of studies. Given the importance of depression, its wide prevalence in the population, and the dysfunction that it causes (both mental and physical), I describe the research findings in a bit more detail. Overall, at least 444 studies have now examined relationships between R/S and depression, dating back to the early 1960s. Of those, 272 (61%) reported significant inverse relationships with depression (including nine studies at a trend level), and 28 (6%) found relationships between R/S and greater depression (including two studies at a trend level). Of the 178 studies with the highest methodological rigor, 119 (67%) reported inverse relationships [124–135] and 13 (7%) found positive relationships with depression [136–148]. 

Of 70 prospective cohort studies, 39 (56%) reported that greater R/S predicted lower levels of depression or faster remission of depression, whereas seven (10%) predicted worse future depression and seven (10%) reported mixed results (both significant positive and negative associations depending on R/S characteristic). Of 30 clinical trials, 19 (63%) found that R/S interventions produced better outcomes than either standard treatment or control groups. Two studies (7%) found standard treatments were superior to R/S interventions [149, 150] and one study reported mixed results.

 Note that an independent review of this literature published in 2003 found that of 147 studies involving 98,975 subjects, the average correlation between R/S and depression was −0.10. Although this is a small correlation, it translates into the same effect size that gender has on depressive symptoms (with the rate of depression being nearly twice as common in women compared to men). Also, the average correlation reported in the 2003 review was 50% stronger in stressed versus nonstressed populations [151].

A widely renowned psychiatric epidemiology group at Columbia University, led by Lisa Miller and Myrna Weissman, has come out with a series of recent reports on R/S and depression studying a cohort of low- and high-risk children born to parents with and without depressive disorder. The findings from this cohort support an inverse link between R/S and depression, particularly in high-risk individuals [152–154]. 

### 4.4. Suicide

Correlations between R/S and suicide attempt, completed suicide, and attitudes toward suicide are consistent with those found for depression, self-esteem, and hope. Those who are depressed, without hope, and with low self-esteem are at greater risk for committing suicide. At least 141 studies have now examined relationships between R/S and the suicide variables above. Of those, 106 (75%) reported inverse relationships and four (3%) found positive relationships. With regard to the 49 studies with the highest methodological rigor, 39 (80%) reported less suicide, fewer suicide attempts, or more negative attitudes toward suicide among the more R/S [155–170] and two (4%) found positive relationships (one study in Delhi, India [171], and one in college students distressed over R/S concerns [172]).

### 4.5. Anxiety

Anxiety and fear often drive people toward religion as a way to cope with the anxiety. Alternatively, R/S may increase anxiety/fear by its threats of punishment for evil deeds and damnation in the next life. There is an old saying that emphasizes this dual role: religion comforts the afflicted and afflicts the comforted. Sorting out cause and effect here is particularly difficult given the few prospective cohort studies that have examined this relationship over time. However, a number of clinical trials have also examined the effects of R/S interventions on anxiety levels. Overall, at least 299 studies have examined this relationship, and of those, 147 (49%) reported inverse association with R/S (three at a trend level), whereas 33 (11%) reported greater anxiety in those who were more R/S. Of the latter, however, only one was a prospective study, one was a randomized clinical trial, and 31 (94%) were cross-sectional studies (where it was not clear whether R/S caused anxiety or whether anxiety increased R/S as a coping response to the anxiety). Of the 67 studies with quality ratings of seven or higher, 38 (55%) reported inverse relationships [173–182] and seven (10%) found positive relationships (greater anxiety among the more R/S) [183–189]. 

Among these 299 studies were 239 cross-sectional studies, 19 prospective cohort studies, 9 single-group experimental studies, and 32 randomized clinical trials. Of the 19 longitudinal studies, 9 (47%) reported that R/S predicted a lower level of anxiety over time; one study (5%) found an increase in anxiety (among women undergoing abortion for fetal anomaly) [189], seven reported no association, and two reported mixed or complex results. Of the nine experimental studies, seven (78%) found a reduction in anxiety following an R/S intervention (before versus after comparison). Of the 32 randomized clinical trials, 22 (69%) reported that an R/S intervention reduced anxiety more than a standard intervention or control condition, whereas one study (3%) found an increase in anxiety following an R/S intervention in persons with severe alcohol dependence [190].

### 4.6. Psychotic Disorder/Schizophrenia

We identified 43 studies that have examined relationships between R/S and chronic psychotic disorders such as schizophrenia. Of the 43 studies examining psychosis, 14 (33%) reported inverse relationships between R/S and psychotic symptoms (one at a trend level), 10 (23%) found a positive relationship between R/S and psychotic symptoms (one at a trend level), eight reported mixed results (significant negative and positive associations, depending on the R/S characteristic measured), and one study reported complex results. Of these studies, seven had quality ratings of seven or higher; of those, two found inverse relationships, two found positive relationship, two reported mixed results (negative and positive), and one found no association. Note that the two studies finding inverse relationships between R/S and psychosis were both prospective studies [191–193], finding that R/S predicted better outcomes in subjects with psychotic disorders or symptoms. Of the two studies reporting positive relationships (both cross-sectional), one study found that importance of religion was significantly and positively associated with religious delusions [194] (not surprising), and the other study found that importance of religion was associated with “psychotic-like” symptoms in a national sample of Mexican Americans [195]; since the latter study involved participants who were not mentally ill, religion-related cultural factors may have influenced this finding. For a recent and more comprehensive discussion of R/S, schizophrenia, other chronic psychotic disorders, and the challenges distinguishing psychotic symptoms from religious beliefs, the reader is referred elsewhere [196]. 

### 4.7. Bipolar Disorder

Despite it's importance and wide prevalence, we could locate only four studies examining the relationship between R/S and bipolar (BP) disorder. Two found a positive association between R/S and bipolar disorder, and the remaining two reported mixed findings (both positive and negative correlations, depending on R/S characteristic). Of the two studies with high-quality ratings, one found a positive association and the other reported mixed findings. The first study of 334 US veterans with BP disorder found that a higher frequency of prayer or meditation was associated with mixed states and a lower likelihood of euthymia, although no association was found between any religious variable and depression or mania [197]. A second study examined a random national sample of 37,000 Canadians and found that those who attributed greater importance to higher spiritual values were more likely to have BP disorder, whereas higher frequency of religious attendance was associated with a lower risk of disorder [198]. In a qualitative study of 35 adults with bipolar disorder (not included in the review above), one of the six themes that participants emphasized when discussing their quality of life was the spiritual dimension. Over one-third of participants in that study talked about the relationship between BP disorder and R/S, emphasizing struggles to disentangle genuine spiritual experiences from the hyperreligiosity of the disorder. In another report, a case of mania precipitated by Eastern meditation was discussed; also included in this article was a review of nine other published cases of psychosis occurring in the setting of meditation [199]. 

### 4.8. Personality Traits

Personality traits most commonly measured today in psychology are the Big Five: extraversion, neuroticism, conscientiousness, agreeableness, and openness to experience. These are assessed by the NEO Personality Inventory [200]. Another personality inventory commonly used in the United Kingdom is the Eysenck Personality Questionnaire, which assesses extraversion, neuroticism, and psychoticism [201]. Relationships between personality traits and R/S using these measures have been examined in many studies [202]. With regard to psychoticism (a trait that assesses risk taking or lack of responsibility, rather than psychotic symptoms), 19 studies have examined its relationship to R/S, with 84% of those reporting significant inverse relationships (and no studies reporting a positive relationship). There have been at least 54 quantitative studies examined relationships between R/S and neuroticism, of which 24% found an inverse relationship and 9% reported a positive relationship (most of the remaining found no association). Concerning extraversion, there have been 50 studies, with 38% reporting a positive relationship with R/S and 6% reporting an inverse or negative relationship. With regard to conscientiousness, there have been 30 studies, of which the majority (63%) reported significant positive relationships with R/S and only 3% found significant inverse relationships. For agreeableness, 30 studies have examined relationships with R/S, and 87% of these studies reported positive relationships (no studies report inverse relationships). Finally, there have been 26 studies examining openness to experience, and of those, 42% found positive relationships with R/S and 12% reported negative relationships. Thus, R/S persons tend to score lower on psychoticism and neuroticism, and higher on extraversion, conscientiousness, agreeableness, and openness to experience. They score especially low on psychoticism and especially high on agreeableness and conscientiousness. These personality traits have physical health consequences that we are only beginning to recognize [203–205]. 

### 4.9. Substance Abuse

If R/S influences one domain of mental health, it is in the area of substance abuse. With regard to alcohol use, abuse, and dependence, at least 278 studies have now examined relationships with R/S. Of those, 240 (86%) reported inverse relationships and only 4 studies (1%) indicated a positive relationship. Of the 145 studies with the best methodology, 131 (90%) reported inverse relationships [206–221] and only one study found a positive relationship [222]. Findings are similar with regard to drug use or abuse. We located 185 studies, of which 84% reported inverse relationship with R/S and only two studies (1%) found positive relationships. Of the 112 best studies, 96 (86%) reported inverse relationships [223–238] and only one study found a positive relationship [239]. The vast majority of these studies are in young persons attending high school or college, a time when they are just starting to establish substance use habits (which for some will interfere with their education, future jobs, family life, and health). Thus, the protective effects of R/S on substance abuse may have influences on health across the lifespan.

### 4.10. Social Problems

Here I examine research in two areas of social instability (delinquency/crime and marital instability) and two areas of social stability (social support and social capital). Given the emphasis that most major world religions place on human relationships, love, and compassion, one might expect that some of the strongest relationships with R/S would be found here, and they are indeed.

#### 4.10.1. Delinquency/Crime

At least 104 studies have examined relationships with R/S. Of those, 82 (79%) reported significant inverse relationships (five at a trend level), whereas three (3%) found positive relationships with more delinquency/crime. Of the 60 studies with quality ratings of 7 or higher, 49 (82%) reported inverse relationships [240–252] and only one study found a positive relationship [253]. Of particular interest are the 10 studies examining relationships between R/S and school grades/performance in adolescents and college students between 2000 and 2009, of which all 10 (100%) found that more R/S youth did better than less religious youth [254].

#### 4.10.2. Marital Instability

We identified 79 studies that examined relationships with marital instability. Of those, 68 (86%) found R/S related to greater marital stability and no studies reported an association with greater marital instability. Of the 38 methodologically most rigorous studies, 35 (92%) reported significant relationships between R/S and greater marital stability [255–265]. An independent meta-analysis reviewing research conducted before the year 2000 likewise concluded that greater religiousness decreased the risk of divorce and facilitated marital functioning and parenting [266].

#### 4.10.3. Social Support

There is substantial evidence indicating a relationship between R/S and social support. Of 74 quantitative peer-reviewed studies of R/S and social support, 61 (82%) found significant positive relationships, and none found inverse relationships. Of the 29 best studies, 27 (93%) reported significant positive relationships [82, 267–274]. For older adults in particular, the most common source of social support outside of family members comes from members of religious organizations [275, 276]. 

#### 4.10.4. Social Capital

Social capital, an indirect measure of community health, is usually assessed by level of community participation, volunteerism, trust, reciprocity between people in the community, and membership in community-based, civic, political, or social justice organizations. Research has examined relationships between R/S and social capital. We located a total of 14 studies, with 11 (79%) finding significant positive relationships between R/S and level of social capital, and none reporting only inverse relationships. Almost all of these studies were of high quality, and of the 13 studies with ratings of seven or higher, 10 (77%) found that R/S was related to greater social capital [277–280]. 

## 5. Explaining the Relationship: R/S and Mental Health

**Figure 2:**
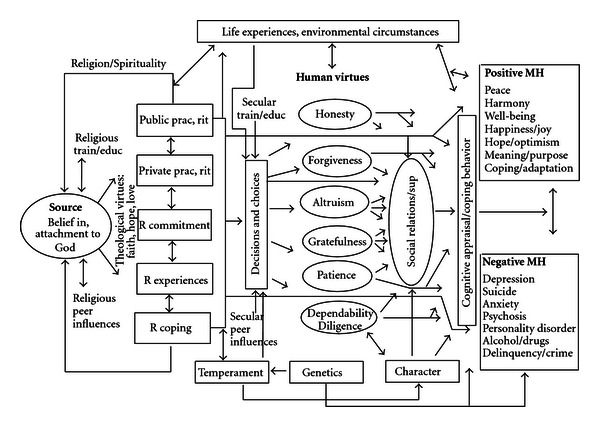
Theoretical model of causal pathways for mental health (MH), based on Western monotheistic religions (Christianity, Judaism, and Islam). (Permission to reprint obtained. Original source: Koenig et al. [17]). For models based on Eastern religious traditions and the Secular Humanist tradition, see elsewhere. (Koenig et al. [24]).

R/S influences mental health through many different mechanisms, although the following are probably the predominant ones (see Figure 2). First, religion provides resources for coping with stress that may increase the frequency of positive emotions and reduce the likelihood that stress will result in emotional disorders such as depression, anxiety disorder, suicide, and substance abuse. Religious coping resources include powerful cognitions (strongly held beliefs) that give meaning to difficult life circumstances and provide a sense of purpose. Religions provide an optimistic worldview that may involve the existence of a personal transcendental force (God, Allah, Jehovah, etc.) that loves and cares about humans and is responsive to their needs. These cognitions also give a subjective sense of control over events (i.e., if God is in control, can influence circumstances, and be influenced by prayer, then prayer by the individual may positively influence the situation). Religious beliefs provide satisfying answers to existential questions, such as “where did we come from,” “why are we here,” and “where are we going,” and the answers apply to both this life and the next life, thus reducing existential angst. These beliefs also help to normalize loss and change and provide role models of persons suffering with the same or similar problems (often illustrated in religious scriptures). Thus, religious beliefs have the potential to influence the cognitive appraisal of negative life events in a way that makes them less distressing. For people with medical illness, these beliefs are particularly useful because they are not lost or impaired with physical disability—unlike many other coping resources that are dependent on health (hobbies, relationships, and jobs/finances).

Second, most religions have rules and regulations (doctrines) about how to live life and how to treat others within a social group. When individuals abide by those rules and regulations, this reduces the likelihood of stressful life events that reduce positive emotions and increased negative ones. Examples of stressful life events that religion may help people avoid are divorce or separation, difficulties with children, financial stress resulting from unfair practices in the marketplace, incarceration for lawbreaking (cheating or crime), and venereal diseases from risky sexual practices. Religions also usually discourage the use of drugs and excessive amounts of alcohol that increases the risk of engaging in the behaviors above (crime, risky sex) that are associated with negative mental health consequences. 

Third, most religions emphasize love of others, compassion, and altruistic acts as well as encourage meeting together during religious social events. These prosocial behaviors have many consequences that buffer stress and lead to human support when support is needed during difficult times. Because religion encourages the helping of others and emphasizes a focus outside of the self, engagement in other-helping activities may increase positive emotions and serve to distract from one's own problems. Religion also promotes human virtues such as honesty, forgiveness, gratefulness, patience, and dependability, which help to maintain and enhance social relationships. The practice of these human virtues may also directly increase positive emotions and neutralize negative ones.

Thus, there are many possible mechanisms by which R/S may enhance mental and social health. This is not to say that R/S always does so. Religion may also be used to justify hatred, aggression, prejudice, and the exclusion of others; gain power and control over vulnerable individuals (as seen in cults); foster rigid thinking and obsessive practices; lead to anxiety, fear, and excessive guilt over minor infractions (and even self-mutilation in some cases); produce psychosocial strains due to failure to live up to high religious standards; lead to escape from dealing with family problems (through excessive involvement in religious or spiritual activities); and delay diagnosis and effective mental health care (due to antagonistic relationships with mental health professionals). While R/S is not a panacea, on the balance, it is generally associated with greater well-being, improved coping with stress, and better mental health. This relationship with mental health has physical health consequences (see Section 7 below).

## 6. Religion, Spirituality, and Health Behaviors

Religious doctrines influence decisions about health and health behaviors. In the Judeo-Christian scriptures, for example, there is an emphasis on caring for the physical body as a “Temple of the Holy Spirit” (see 1 Corinthian 6:19-20) [281]. Religious scriptures in other faith traditions also emphasize the person's responsibility to care for and nourish their physical body [282–284]. Behaviors that have the potential to harm the body are usually discouraged. This is reflected in teachings from the pulpit and influences what is considered appropriate within religious social groups. In summarizing the research on R/S and health behaviors, I cite only a few of the studies with high-quality ratings since there are so many.

### 6.1. Cigarette Smoking

The influence of R/S is most evident in it's “effects” on cigarette smoking. At least 137 studies have examined relationship between R/S and smoking, and of those, 123 (90%) reported statistically significant inverse relationships (including three at a trend level) and no studies found either a significant or even a trend association in the other direction. Of the 83 methodologically most rigorous studies, 75 (90%) reported inverse relationships with R/S involvement [213, 285–294]. Not surprisingly, the physical health consequences of not smoking are enormous. Decreased cigarette smoking will mean a reduction in chronic lung disease, lung cancer, all cancers (30% being related to smoking), coronary artery disease, hypertension, stroke, and other cardiovascular diseases.

### 6.2. Exercise

Level of exercise and physical activity also appears linked to R/S. We located 37 studies that examined this relationship. Of those, 25 (68%) reported significant positive relationships (two at a trend level) between R/S involvement and greater exercise or physical activity, whereas six (16%) found significant inverse relationships. Of 21 studies with the highest quality ratings, 16 (76%) reported positive associations [82, 295–300] and two (10%) found negative associations [296, 301].

Writers in the popular press have encouraged the combining of R/S activity and exercise through “prayer walking” [302, 303] and “walking meditation.” [304].

### 6.3. Diet

At least 21 studies have examined relationships between R/S and a healthy diet. A healthy diet here involves increased intake of fiber, green vegetables, fruit, and fish; low intake of snacks, processed foods, and fat; regular vitamin intake; frequent eating of breakfast; overall better nutrition (following recommended nutritional guidelines). Of those studies, 13 (62%) found a significant positive association between R/S and a healthier diet (one at a trend level) and one found a worse diet [305]. Among the 10 studies with the highest quality ratings, seven (70%) reported a better diet among those who were more R/S [213, 306–310]. In addition, we identified 23 studies that examined relationships between R/S and blood cholesterol levels. Of those, more than half (12 studies) found significantly lower cholesterol among those who were more R/S, whereas three studies (13%) reported significantly higher cholesterol levels. Of the nine best studies, five (56%) reported lower cholesterol [311–313] or a lowering of cholesterol in response to a R/S intervention [314, 315], whereas one found higher cholesterol (but only in Mexican American men) [316].

### 6.4. Weight

Although R/S people tend to eat a healthier diet, they also eat more of it. This, then, is the one health behavior that places R/S individuals at greater risk for medical illness. At least 36 studies have examined the associations between weight (or body mass index) and R/S involvement. Of those, 14 (39%) found a positive relationship (R/S associated with greater weight), whereas only seven (19%) reported an inverse relationship. The situation does not improve when results from the most rigorously designed studies are examined. Among the 25 studies with the highest quality ratings, 11 (44%) reported greater weight among the more R/S [82, 317–322] and five (20%) found lower weight (or less underweight [323]). Lower weight among the more R/S appears only in a few religious groups (Amish [324], Jews [325], and Buddhists [326]), in those with certain demographic characteristics (white, older, and high education) [327], and in response to a specific R/S intervention [328] or practice [314, 329]. Faith-based weight-reduction programs in religious communities have been shown to be effective [328, 330, 331].

### 6.5. Sexual Behavior

We identified 95 studies that examined relationships between R/S and risky sexual activity (sex outside of marriage, multiple partners, etc.). Of those, 82 studies (86%) found significant inverse relationships with R/S (one at a trend level) and only one study (1%) found a significant relationship with more risky sexual activity [332]. Of the 50 highest quality studies, 42 (84%) reported inverse relationships [333–343] and none found a positive one. If those who are more R/S engage in less risky sexual behavior, this means they should have fewer venereal diseases, that is, less syphilis, gonorrhea, herpes, chancroid, chlamydia, viral hepatitis, and human papillomavirus and human immunodeficiency virus, many of which have serious physical health consequences.

## 7. Religion, Spirituality, and Physical Health

There is rapidly growing evidence that stress and negative emotions (depression, anxiety) have (1) adverse effects on physiological systems vital for maintenance of physical health and healing [344–346], (2) increase susceptibility to or worse outcomes from a wide range of physical illnesses [347–351], and (3) may shorten the lifespan prematurely [352, 353]. Social support, in turn, has long been known to protect against disease and increase longevity [354–356]. By reducing stress and negative emotions, increasing social support, and positively affecting health behaviors, R/S involvement should have a favorable impact on a host of physical diseases and the response of those diseases to treatment. As in the earlier sections, I cite high-quality studies as examples. Since there are fewer high-quality studies for physical health than for mental health or for health behaviors, I cite all of the studies with ratings of seven or higher.

### 7.1. Coronary Heart Disease (CHD)

Given the strong connections between psychosocial stressors, health behaviors, and CHD, it is not surprising that there is a link with R/S. Our review uncovered 19 studies that examined associations between R/S and CHD. Of those, 12 (63%) reported a significant inverse relationship, and one study reported a positive relationship. Of the 13 studies with the most rigorous methodology, nine (69%) found inverse relationships with CHD [357–365] and one found a positive one [366]. In addition, there have been at least 16 studies examining relationships between R/S and cardiovascular reactivity, heart rate variability, outcomes following cardiac surgery, and other cardiovascular functions. Of those, 11 studies (69%) reported that R/S was significantly related to positive cardiovascular functions or outcomes [367–374] or to lower levels of inflammatory markers such as C-reactive protein [375–377] and fibrinogen [378] that place individuals at high risk for cardiovascular disease. 

### 7.2. Hypertension

The word “hypertension” itself suggests a relationship with stress or tension, and high blood pressure has been linked to greater psychosocial stress [379–381]. At least 63 studies have examined the relationship between R/S and blood pressure (BP), of which 36 (57%) reported significantly lower BP in those who are more R/S (five at a trend level) and seven (11%) reported significantly higher BP (one at a trend level). Of the 39 highest quality studies, 24 (62%) report lower BP (including one at a trend level) among those who are more R/S [382–394] or in response to an R/S intervention [328, 395–404] (including a study whose results were reported twice, once for the overall sample and once for the sample stratified by race).

Two lower quality studies [405, 406] and five well-done studies [407–411] (13%, including one at a trend level), however, reported higher BP in the more R/S or with religious fasting. The reason for an association between R/S and higher BP is not entirely clear. Perhaps, in certain population subgroups, intrapsychic religious conflict between psychosexual drives and religious standards creates unconscious stress that elevates BP. However, there is another possibility. This may be related to confounding by ethnicity. Three of the five studies reporting increased BP with increased R/S included in their samples a large proportion of ethnic minorities (samples from large urban settings such as Detroit and Chicago, made up of 36% to 100% African Americans). Since African Americans are more likely to have high BP (40% with hypertension) [412] and because African Americans are also the most religious ethnic group in society [413], it may be that controlling for race in these analyses is simply not sufficient to overcome this powerful confound.

### 7.3. Cerebrovascular Disease

Relationships between R/S, hypertension and other cardiovascular diseases or disease risk factors ought to translate into a lower risk of stroke. We located nine studies that examined this relationship, of which four reported a lower risk of stroke, all having quality ratings of seven or higher [414–417]. 

One study, however, reported significantly more carotid artery thickening, placing R/S individuals at higher risk for stroke [418]. Again, however, 30% of that sample was African American an ethnic group, known to be both highly religious and at high risk for stroke.

### 7.4. Alzheimer's Disease and Dementia

Physiological changes that occur with stress and depression (elevated blood cortisol, in particular) are known to adversely affect the parts of the brain responsible for memory [419–421]. The experience of negative emotions may be like pouring hydrochloric acid on the brain's memory cells [422]. By reducing stress and depression through more effective coping, R/S may produce a physiological environment that has favorable effects on cognitive functioning. Furthermore, R/S involvement may also engage higher cortical functions involved in abstract thinking (concerning moral values or ideas about the transcendent) that serve to “exercise” brain areas necessary for retention of memories. Regardless of the mechanism, at least 21 studies have examined relationships between R/S involvement and cognitive function in both healthy persons and individuals with dementia. Of those, 10 (48%) reported significant positive relationships between R/S and better cognitive functioning and three (14%) found significant negative relationships. Of the 14 studies with the highest quality ratings, eight (57%) reported positive relationships [423–430] and three (21%) reported negative relationships with cognitive function [431–433]. Studies finding negative relationships between R/S and cognitive function may be due to the fact that R/S persons have longer lifespans (see below), increasing the likelihood that they will live to older ages when cognition tends to decline. More recent research supports a positive link between R/S and better cognitive function in both dementia and in old age [434, 435]. 

### 7.5. Immune Function

Intact immune function is critical for health maintenance and disease prevention and is assessed by indicators of cellular immunity, humoral immunity, and levels of pro- and anti-inflammatory cytokines. We identified 27 studies on relationships between R/S and immune functions, of which 15 (56%) found positive relationships or positive effects in response to a R/S intervention, and one (4%) found a negative effect [436]. Of the 14 studies with the highest quality ratings, 10 (71%) reported significant positive associations [437–443] or increased immune functions in response to a R/S intervention [444–447]. No high-quality study found only an inverse association or negative effect, although one study reported mixed findings [448]. In that study, religious attendance was related to significantly poorer cutaneous response to antigens; however, it was also related (at a trend level) to higher total lymphocyte count, total T-cell count, and helper T-cell count. In addition, importance of religious or spiritual expression was related to significantly higher white blood cell count, total lymphocyte count, total T cells, and cytotoxic T cell activity.

There have also been a number of studies examining R/S and susceptibility to infection (or viral load in those with HIV), which could be considered an indirect measure of immune function. We identified 12 such studies, of which eight (67%) reported significantly lower infection rates or lower viral loads in those who were more R/S (including one at a trend level); none found greater susceptibility to infection or greater viral load. Ten of the 12 studies had quality ratings of 7 or higher; of those, seven (70%) reported significant inverse associations with infection/viral load [440, 441, 449–454].

### 7.6. Endocrine Function

Because stress hormones (cortisol, epinephrine, and norepinephrine) have a known influence on immune (and cardiovascular) functions, they are important factors on the pathway between R/S involvement and health [455, 456]. We identified 31 studies that examined R/S and associations with or effects on endocrine functions. Of those, 23 (74%) reported positive relationships or positive effects and no studies reported negative associations or negative effects. Of the 13 methodologically most rigorous studies, nine (69%) reported positive associations with R/S [457–461] or positive effects of an R/S intervention (all involving Eastern meditation) [462–465]. We (at Duke) are currently examining the effects of religious cognitive-behavioral therapy on a host of pro- and anti-inflammatory cytokines, cortisol, and catecholamines in patients with major depressive disorder, although results will not be available until 2014 [466]. 

### 7.7. Cancer

At least 29 studies have examined relationships between R/S and either the onset or the outcome of cancer (including cancer mortality). Of those, 16 (55%) found that those who are more R/S had a lower risk of developing cancer or a better prognosis, although two (7%) reported a significantly worse prognosis [467, 468]. Of the 20 methodologically most rigorous studies, 12 (60%) found an association between R/S and lower risk or better outcomes [469–480], and none reported worse risk or outcomes. The results from some of these studies can be partially explained by better health behaviors (less cigarette smoking, alcohol abuse, etc.), but not all. Effects not explained by better health behaviors could be explained by lower stress levels and higher social support in those who are more R/S. Although cancer is not thought to be as sensitive as cardiovascular disorders to psychosocial stressors, psychosocial influences on cancer incidence and outcome are present (discussions over this are ongoing [481, 482]).

### 7.8. Physical Functioning

Ability to function physically, that is, performing basic and instrumental activities of daily living such as toileting, bathing, shopping, and using a telephone, is a necessary factor for independent living. Persons who are depressed, unmotivated, or without hope are less likely to make attempts to maintain their physical functioning, particularly after experiencing a stroke or a fall that forces them into a rehabilitation program to regain or compensate for their losses. Several studies have examined the role that R/S plays in helping people to maintain physical functioning as they grow older or regain functioning after an illness. We identified 61 quantitative studies that examined relationships between R/S and disability level or level of functioning. Of those, 22 (36%) reported better physical functioning among those who were more R/S, 14 (23%) found worse physical functioning, and six studies reported mixed findings. Considering the 33 highest quality studies, 13 (39%) reported significantly better physical functioning among those who were more R/S (including one study at a trend level) [483–495], six (18%) found worse functioning [496–501], and five studies (15%) reported mixed results [82, 124, 502–504] (significant positive and negative associations, depending on R/S characteristic). Almost all of these studies involve self-reported disability and many were cross-sectional, making it impossible to determine order of causation—that is, (1) does R/S prevent the development of disability, (2) does disability prevent R/S activity, (3) does R/S promote disability, or (4) does disability cause people to turn to religion to cope with disability.

### 7.9. Self-Rated Health

There is more agreement across studies regarding the relationship between R/S and self-rated health (SRH) than between R/S and physical functioning. While based on participants' subjective impression, self-rated health is strongly related to objective health, that is, future health, health services use, and mortality [505–507]. Might R/S, perhaps because it is related to greater optimism and hope, influence one's self-perceptions of health in a positive way? At least 50 studies have now examined the relationship between R/S and self-rated health. Of those, 29 (58%) reported that R/S was related to better SRH, while five (10%) found that it was related to worse SRH. Of the 37 methodologically most rigorous studies, 21 (57%) reported significant positive relationships between R/S and SRH [503, 508–527], whereas three (8%) found the opposite [528–530]. 

### 7.10. Pain and Somatic Symptoms

On the one hand, pain and other distressing somatic symptoms can motivate people to seek solace in religion through activities such as prayer or Scripture study. Thus, R/S is often turned to in order to cope with such symptoms. For example, in an early study of 382 adults with musculoskeletal complains, R/S coping was the most common strategy for dealing with pain and was considered the second most helpful in a long list of coping behaviors [531]. More recent research supports this earlier report [532]. On the other hand, R/S may somehow cause an increase in pain and somatic symptoms, perhaps by increasing concentration on negative symptoms or through the physical manifestations of hysteria, as claimed by Charcot in his copious writings around the turn of the 20th century [533].

We identified 56 studies that examined relationships between R/S and pain. Of those, 22 (39%) reported inverse relationships between R/S and pain or found benefits from an R/S intervention, whereas 14 (25%) indicated a positive relationship between R/S and greater pain levels (13 of 14 being cross-sectional). Of the 18 best studies, nine (50%) reported inverse relationships (less pain among the more R/S [534] or reduced pain in response to a R/S intervention [535–542]), while three (20%) reported positive relationships (worse pain in the more R/S) [543–545]. Research suggests that meditation is particularly effective in reducing pain, although the effects are magnified when a religious word is used to focus attention [546, 547]. No clinical trials, to my knowledge, have shown that meditation or other R/S interventions increase pain or somatic symptoms.

### 7.11. Mortality

The most impressive research on the relationship between R/S and physical health is in the area of mortality. The cumulative effect of R/S, if it has any benefits to physical health, ought to reveal itself in an effect on mortality. The research suggests it does. At least 121 studies have examined relationships between R/S and mortality. Most of these are prospective cohort studies, where baseline R/S is assessed as a predictor of mortality during the observation period, controlling for confounders. Of those studies, 82 (68%) found that greater R/S predicted significantly greater longevity (three at a trend level), whereas six studies (5%) reported shorter longevity. Considering the 63 methodologically most rigorous studies (quality ratings of 8 or higher), 47 (75%) found R/S predicting greater longevity (two at trend level) [548–566], whereas three (5%) reported shorter longevity [567–569]. Another systematic review [570] and two meta-analyses [571, 572] have confirmed this relationship between R/S and longer survival. The effects have been particularly strong for frequency of attendance at religious services in these three reviews. Survival among frequent attendees was increased on average by 37%, 43%, and 30% (mean effect being 37% across these reviews). An increased survival of 37% is highly significant and equivalent to the effects of cholesterol lowering drugs or exercise-based cardiac rehabilitation after myocardial infarction on survival [573]. 

## 8. Explaining the Relationship: R/S and Physical Health

**Figure 3:**
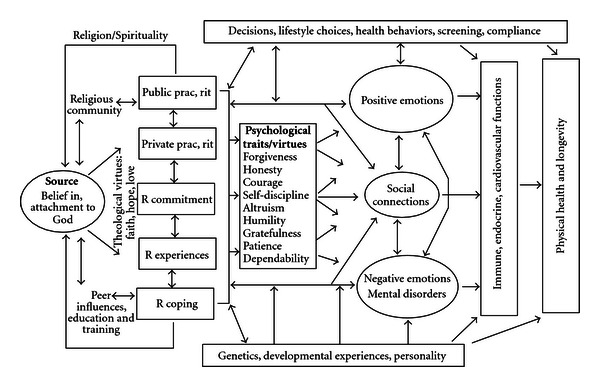
Theoretical model of causal pathways to physical health for Western monotheistic religions (Christianity, Islam, and Judaism). (Permission to reprint obtained. Original source: Koenig et al. [17]). For models based on Eastern religious traditions and the Secular Humanist tradition, see elsewhere (Koenig et al. [24]).

How might R/S involvement influence physical health and longevity? There are at least three basic pathways: psychological, social, and behavioral (see Figure 3).

### 8.1. Psychological

As noted above, there is ample evidence that R/S—because it facilitates coping and imbues negative events with meaning and purpose—is related to better mental health (less depression, lower stress, less anxiety, greater well-being, and more positive emotions). Furthermore, several randomized clinical trials have shown that R/S interventions improve mental health (at least in those who are R/S). There is also much evidence that poor mental health has adverse physiological consequences that worsen physical health and shorten the lifespan (see earlier references). Thus, it stands to reason that R/S might influence physical health through psychological pathways.

### 8.2. Social

R/S involvement is associated with greater social support, greater marital stability, less crime/delinquency, and greater social capital. R/S beliefs and doctrines encourage the development of human virtues such as honesty, courage, dependability, altruism, generosity, forgiveness, self-discipline, patience, humility, and other characteristics that promote social relationships. Participation in a R/S community not only provides supportive social connections and opportunities for altruism (through volunteering or other faith-based altruistic activities), but also increases the flow of health information that may increase disease screening and promote health maintenance. Social factors, in turn, are known to influence both mental health and physical health and predict greater longevity [574–576]. Again, if R/S boosts supportive social interactions and increases community trust and involvement, then it should ultimately influence physical health as well.

### 8.3. Health Behaviors

Finally, R/S promotes better health behaviors, and is associated with less alcohol and drug use, less cigarette smoking, more physical activity and exercise, better diet, and safer sexual practices in the overwhelming majority of studies that have examined these relationships. Living a healthier lifestyle will result in better physical health and greater longevity. Consider the following report that appeared on CNN (Cable Network News). On January 3, 2009, after the death of the Guinness Book of World Records' oldest person, Maria de Jesus age 115, next in line was Gertrude Baines from Los Angeles. Born to slaves near Atlanta in 1894, she was described at 114 years old as “spry,” “cheerful,” and “talkative.” When she was 112 years old, Ms. Baines was asked by a CNN correspondent to explain why she thought she had lived so long. Her reply: “God. Ask Him. I took good care of myself, the way he wanted me to.” Brief and to the point.

### 8.4. Other Pathways

There are many ways by which R/S could have a positive influence on physical health, although the pathways above are probably the major ones. Genetic and developmental factors could also play a role in explaining these associations. There is some evidence that personality or temperament (which has genetic roots) influences whether or not a person becomes R/S. To what extent R/S persons are simply born healthier, however, is quite controversial. Note that more R/S persons are typically those with the least resources (minority groups, the poor, and the uneducated), both in terms of finances and access to healthcare resources. Karl Marx said that religion is the “opiate of the masses.” Rather than being born healthier, then, the opposite is more likely to be true for R/S persons. R/S could actually be viewed as acting counter to an evolutionary force that is trying to weed genetically vulnerable people from the population. R/S involvement is providing the weak with a powerful belief system and a supportive community that enables them to survive. For a more complete discussion of the role of genetic factors in the R/S-physical health relationship, see the *Handbook* [577].

Another important point needs to be made. Nowhere do I claim that supernatural mechanisms are responsible for the relationship between R/S and health. The pathways by which R/S influences physical health that researchers can study using the natural methods of science must be those that exist within nature—that is, psychological, social, behavioral, and genetic influences. Thus, this research says nothing about the existence of supernatural or transcendent forces (which is a matter of faith), but rather asks whether belief in such forces (and the behaviors that result from such beliefs) has an effect on health. There is every reason to think it does.

## 9. Clinical Implications

There are clinical implications from the research reviewed above that could influence the way health professionals treat patients in the hospital and clinic.

### 9.1. Rationale for Integrating Spirituality

There are many practical reasons why addressing spiritual issues in clinical practice is important. Here are eight reasons [578] (and these are not exhaustive).

First, many patients are R/S and have spiritual needs related to medical or psychiatric illness. Studies of medical and psychiatric patients and those with terminal illnesses report that the vast majority have such needs, and most of those needs currently go unmet [579, 580]. Unmet spiritual needs, especially if they involve R/S struggles, can adversely affect health and may increase mortality independent of mental, physical, or social health [581].

Second, R/S influences the patient's ability to cope with illness. In some areas of the country, 90% of hospitalized patients use religion to enable them to cope with their illnesses and over 40% indicate it is their primary coping behavior [582]. Poor coping has adverse effects on medical outcomes, both in terms of lengthening hospital stay and increasing mortality [583].

Third, R/S beliefs affect patients' medical decisions, may conflict with medical treatments, and can influence compliance with those treatments. Studies have shown that R/S beliefs influence medical decisions among those with serious medical illness [584, 585] and especially among those with advanced cancer [586] or HIV/AIDs [587].

Fourth, physicians' own R/S beliefs often influence medical decisions they make and affect the type of care they offer to patients, including decisions about use of pain medications [588], abortion [589], vaccinations [590], and contraception [591]. Physician views about such matters and how they influence the physician's decisions, however, are usually not discussed with a patient.

Fifth, as noted earlier, R/S is associated with both mental and physical health and likely affects medical outcomes. If so, then health professionals need to know about such influences, just as they need to know if a person smokes cigarettes or uses alcohol or drugs. Those who provide health care to the patient need to be aware of all factors that influence health and health care.

Sixth, R/S influences the kind of support and care that patients receive once they return home. A supportive faith community may ensure that patients receive medical followup (by providing rides to doctors' offices) and comply with their medications. It is important to know whether this is the case or whether the patient will return to an apartment to live alone with little social interaction or support.

Seventh, research shows that failure to address patients' spiritual needs increases health care costs, especially toward the end of life [592]. This is a time when patients and families may demand medical care (often very expensive medical care) even when continued treatment is futile. For example, patients or families may be praying for a miracle. “Giving up” by withdrawing life support or agreeing to hospice care may be viewed as a lack of faith or lack of belief in the healing power of God. If health professionals do not take a spiritual history so that patients/families feel comfortable discussing such issues openly, then situations may go on indefinitely and consume huge amounts of medical resources.

Finally, standards set by the Joint Commission for the Accreditation of Hospital Organizations (JCAHO) and by Medicare (in the US) require that providers of health care show respect for patients' cultural and personal values, beliefs, and preferences (including religious or spiritual beliefs) [593]. This point was reinforced by a personal communication with Doreen Finn (dfinn@jointcommission.org), Senior Associate Director, who works under Mark Pelletier (mpelletier@jointcommission.org), Executive Director, JCAHO, Hospital Accreditation (January 6–12, 2012). If health professionals are unaware of those beliefs, they cannot show respect for them and adjust care accordingly.

### 9.2. How to Integrate Spirituality into Patient Care

What would I recommend in terms of addressing spiritual issues in clinical care?

First and foremost, health professionals should take a brief spiritual history. This should be done for all new patients on their first evaluation, especially if they have serious or chronic illnesses, and when a patient is admitted to a hospital, nursing home, home health agency, or other health care setting. The purpose is to learn about (1) the patient's religious background, (2) the role that R/S beliefs or practices play in coping with illness (or causing distress), (3) beliefs that may influence or conflict with decisions about medical care, (4) the patient's level of participation in a spiritual community and whether the community is supportive, and (5) any spiritual needs that might be present [594]. It is the *health professional*, not the chaplain, who is responsible for doing this two-minute “screening” evaluation. If spiritual needs are discovered, then the health professional would make a referral to pastoral care services so that the needs can be addressed. The spiritual history (and any spiritual needs addressed by pastoral services) should be documented in the medical record so that other health professionals will know that this has been done. Although notes need not be detailed, enough information should be recorded to communicate essential issues to other hospital staff.

Ideally, the physician, as head of the medical care team, should take the spiritual history. However, since only about 10% of physicians in the US “often or always” do so [595], the task often falls to the nurse or to the social worker. Although systematic research is lacking in this area, most nurses and social workers do not take a spiritual history either. Simply recording the patient's religious denomination and whether they want to see a chaplain, the procedure in most hospitals today, is NOT taking a spiritual history.

Second, R/S beliefs of patients uncovered during the spiritual history should always be respected. Even if beliefs conflict with the medical treatment plan or seem bizarre or pathological, the health professional should not challenge those beliefs (at least not initially), but rather take a neutral posture and ask the patient questions to obtain a better understanding of the beliefs. Challenging patients' R/S beliefs is almost always followed by resistance from the patient, or quiet noncompliance with the medical plan. Instead, the health professional should consult a chaplain and either follow their advice or refer the patient to the chaplain to address the situation. If the health professional is knowledgeable about the patient's R/S beliefs and the beliefs appear generally healthy, however, it would be appropriate to actively support those beliefs and conform the healthcare being provided to accommodate the beliefs.

Third, most health professionals without clinical pastoral education do not have the skills or training to competently address patients' spiritual needs or provide advice about spiritual matters. Chaplains have extensive training on how to do this, which often involves years of education and experience addressing spiritual issues. They are the true experts in this area. For any but the most simple spiritual needs, then, patients should be referred to chaplains to address the problem.

Fourth, conducting a spiritual history or contemplating a spiritual intervention (supporting R/S beliefs, praying with patients) should always be patient centered and patient desired. The health professional should never do anything related to R/S that involves coercion. The patient must feel in control and free to reveal or not reveal information about their spiritual lives or to engage or not engage in spiritual practices (i.e., prayer, etc.). In most cases, health professionals should not ask patients if they would like to pray with them, but rather leave the initiative to the patient to request prayer. The health professional, however, may inform R/S patients (based on the spiritual history) that they are open to praying with patients if that is what the patient wants. The patient is then free to initiate a request for prayer at a later time or future visit, should they desire prayer with the health professional. If the patient requests, then a short supportive prayer may be said aloud, but quietly, with the patient in a private setting. Before praying, however, the health professional should ask the patient what he or she wishes prayer for, recognizing that every patient will be different in this regard. Alternatively, the clinician may simply ask the patient to say the prayer and then quietly confirm it with an “amen” at the end.

Fifth, R/S beliefs of health professionals (or lack of belief) should not influence the decision to take a spiritual history, respect and support the R/S beliefs of patients, or make a referral to pastoral services. These activities should always be patient centered, not centered on the health professional. One of the most common barriers to addressing spiritual issues is health professionals' discomfort over discussing such issues. This often results from lack of personal R/S involvement and therefore lack of appreciation for the importance and value of doing so. Lack of comfort and understanding should be overcome by training and practice. Today, nearly 90% of medical schools (and many nursing schools) in the US include something about R/S in their curricula [596] and this is also true to a lesser extent in the United Kingdom [597] and Brazil [598]. Thus, spirituality and health is increasingly being addressed in medical and nursing training programs.

Sixth, health professionals should learn about the R/S beliefs and practices of different religious traditions that relate to healthcare, especially the faith traditions of patients they are likely to encounter in their particular country or region of the country. There are many such beliefs and practices that will have a direct impact on the type of care being provided, especially when patients are hospitalized, seriously ill or near death. A brief description of beliefs and practices for health professionals related to birth, contraception, diet, death, and organ donation is provided elsewhere [599].

Finally, if spiritual needs are identified and a chaplain referral is initiated, then the health professional making the referral is responsible for following up to ensure that the spiritual needs were adequately addressed by the chaplain. This is especially true given the impact that unmet spiritual needs are likely to have on both medical outcomes and healthcare costs. Given the short lengths of stay in today's modern hospital (often only 2–4 days), spiritual needs identified on admission are unlikely to be resolved by discharge. Therefore, a spiritual care discharge plan will need to be developed by the hospital social worker in consultation with the chaplain, which may involve (with the patient's written consent) contact with the patient's faith community to ensure that spiritual needs are addressed when the patient returns home. In this way, continuity of pastoral care will be ensured between hospital and community.

## 10. Conclusions

Religious/spiritual beliefs and practices are commonly used by both medical and psychiatric patients to cope with illness and other stressful life changes. A large volume of research shows that people who are more R/S have better mental health and adapt more quickly to health problems compared to those who are less R/S. These possible benefits to mental health and well-being have physiological consequences that impact physical health, affect the risk of disease, and influence response to treatment. In this paper I have reviewed and summarized hundreds of quantitative original data-based research reports examining relationships between R/S and health. These reports have been published in peer-reviewed journals in medicine, nursing, social work, rehabilitation, social sciences, counseling, psychology, psychiatry, public health, demography, economics, and religion. The majority of studies report significant relationships between R/S and better health. For details on these and many other studies in this area, and for suggestions on future research that is needed, I again refer the reader to the *Handbook of Religion and Health* [600].

The research findings, a desire to provide high-quality care, and simply common sense, all underscore the need to integrate spirituality into patient care. I have briefly reviewed reasons for inquiring about and addressing spiritual needs in clinical practice, described how to do so, and indicated boundaries across which health professionals should not cross. For more information on how to integrate spirituality into patient care, the reader is referred to the book, *Spirituality in Patient Care* [601]. The field of religion, spirituality, and health is growing rapidly, and I dare to say, is moving from the periphery into the mainstream of healthcare. All health professionals should be familiar with the research base described in this paper, know the reasons for integrating spirituality into patient care, and be able to do so in a sensible and sensitive way. At stake is the health and well-being of our patients and satisfaction that we as health care providers experience in delivering care that addresses the whole person—body, mind, and spirit.
